# Performance of baseline FDG-PET/CT radiomics for prediction of bone marrow minimal residual disease status in the LyMa-101 trial

**DOI:** 10.1038/s41598-023-45215-y

**Published:** 2023-10-24

**Authors:** Caroline Bodet-Milin, Cyrille Morvant, Thomas Carlier, Gauthier Frecon, Olivier Tournilhac, Violaine Safar, Françoise Kraeber-Bodere, Steven Le Gouill, Elizabeth Macintyre, Clément Bailly

**Affiliations:** 1 Université de Nantes, CHU Nantes, CNRS, Inserm, CRCINA, 44000 Nantes, France; 2 Nuclear Medicine Unit, University Hospital, 44093 Nantes, France; 3 Haematology and Cell Therapy Department, Hôpital Estaing, CHU de Clermont-Ferrand, Clermont-Ferrand, France; 4 Department of Hematology, Hospices Civils de Lyon, Lyon Sud Hospital, Pierre-Bénite, France; 5 Institut Curie, Paris and Saint-Cloud, Université Versailles-Saint Quentin, Saint-Cloud, France; 6 Onco-Haematology, Université de Paris, Hôpital and Institut Necker-Enfants Malades, Assistance-Publique-Hôpitaux de Paris, INSERM U1151, Paris, France

**Keywords:** Cancer imaging, Biomarkers, Lymphoma

## Abstract

The prognostic value of ^18^F-Fluorodeoxyglucose positron emission tomography/computed tomography (FDG-PET/CT) at baseline or the predictive value of minimal residual disease (MRD) detection appear as potential tools to improve mantle cell lymphoma (MCL) patients’ management. The LyMa-101, a phase 2 trial of the LYSA group (ClinicalTrials.gov:NCT02896582) reported induction therapy with obinutuzumab, a CD20 monoclonal antibody. Herein, we investigated the added prognostic value of radiomic features (RF) derived from FDG-PET/CT at diagnosis for MRD value prediction. FDG-PET/CT of 59 MCL patients included in the LyMa-101 trial have been independently, blindly and centrally reviewed. RF were extracted from the disease area with the highest uptake and from the total metabolic tumor volume (TMTV). Two models of machine learning were used to compare several combinations for prediction of MRD before autologous stem cell transplant consolidation (ASCT). Each algorithm was generated with or without constrained feature selections for clinical and laboratory parameters. Both algorithms showed better discrimination performances for negative vs positive MRD in the lesion with the highest uptake than in the TMTV. The constrained use of clinical and biological features showed a clear loss in sensitivity for the prediction of MRD status before ASCT, regardless of the machine learning model. These data plead for the importance of FDG-PET/CT RF compared to clinical and laboratory parameters and also reinforced the previously made hypothesis that the prognosis of the disease in MCL patients is linked to the most aggressive contingent, within the lesion with the highest uptake.

## Introduction

The management of mantle cell lymphoma (MCL) has dramatically progressed with the use of autologous stem cell transplant consolidation (ASCT) and new targeted therapies^1^. The predictive value of minimal residual disease (MRD) detection, measuring the deepness of treatment response^2,3^ and the prognostic value of ^18^F-Fluorodeoxyglucose positron emission tomography/computed tomography (FDG-PET/CT) at baseline^4^ appear as additional tools to improve MCL patients’ management. A growing number of studies have focused on the extraction of a large amount of quantitative data from multimodal medical images, often referred to as "textural features" or "radiomics". A few studies have successfully explored the potential combination of these metrics with clinical data in MCL patients^5,6^. As shown in the prospective LyMA-PET study and supported in a work by Mayerhoefer et al., combining radiomic features (RF) with bio-clinical scores can potentially improve risk stratification. All of these efforts provide the groundwork for future studies exploring the value of RF in PET imaging. In this regard, the multicentric LyMa-101, a phase 2 trial of the LYSA group (ClinicalTrials.gov, NCT02896582) reported the success of an induction therapy with obinutuzumab plus DHAP (dexamethasone, high-dose cytarabine, cisplatin), on MRD status in the bone marrow after four cycles^7^. In the LyMa-101 study, obinutuzumab appeared as a valuable antiCD20 antibody alternative to Rituximab in patients with mantle-cell lymphoma who are eligible for frontline transplantation. This work has also highlighted the interest of early surrogate markers, such as MRD to detect patients responding poorly to chemotherapy and who might therefore benefit from alternative approaches at a much earlier stage. The aim of our study was to investigate in this cohort the potential added prognostic value of RF derived from FDG-PET/CT at diagnosis for MRD prediction at the end of induction.

## Materials and methods

Baseline FDG-PET/CT of MCL patients were retrieved and independently, blindly and centrally reviewed. FDG-PET/CT images were acquired according to local protocols and following the rules of good practice. FDG-PET/CT images were analysed on a dedicated workstation (PLANET®Onco-Solution, Dosisoft, France). The Fuzzy Local Adaptive Bayesian (FLAB) approach was used for volume segmentation^8^. RF were extracted from the disease area with the highest uptake and from the total metabolic tumor volume (TMTV) using the IBSI compliant PyRadiomics framework with two different methods of quantization (linear using 64 bins and absolute using fixed bins of 0.3 SUV). The first step was dedicated to select the most robust RF with respect to randomness. For this purpose, RF were first tested by shuffling 50 times the signal within the disease area segmentation and non-contributive ones (i.e. identical results within 2 × 1,96σ over 50 iterations) were excluded^9^. In a second step, RF (including the conventional FDG-PET/CT features: SUVmax, total metabolic volume, total lesion glycolysis and SUVmax gradient (calculated as the difference between SUVmax and the pathological focus with minimal activity for each patient)) were further selected using the Maximum-Relevance-Minimum-Redundancy (MRMR) and normalized taking into account outliers. Two models of machine learning (support vector machine (SVC) and Ridge logistic regression (LR Ridge)) were assessed. A leave-one-out cross validation (LOOCV) on the training set was performed to select the hyperparameters. Several combinations for prediction of bone marrow MRD before ASCT were considered. Briefly, each algorithm was generated with or without constrained feature selections for clinical and laboratory parameters (age, sex, ECOG performance status, White Blood Count (WBC), Lactate Deshydrogenase level (LDH), MIPI score, Ann Arbor stage and blastoid variant)^10^. This step was repeated using the ten most important RF according to MRMR and extracted from the disease area with the highest uptake and from the TMTV. Consequently, four different models were analysed. The discrimination performance of the models was evaluated by comparing the areas under receiver operator characteristic (ROC) curves (AUC). The imbalanced datasets property was considered through the use of SMOTENC and the pipeline was repeated five times to derive the resuls variability. SMOTENC (Synthetic Minority Over-sampling Technique for Nominal and Continuous variables) is an extension of SMOTE for dataset containing continuous and categorical variables. The aim is to handle the minority class by oversampling in the context of imbalance dataset. The study was approved by Ouest VI (Brest, France) Ethics Committee, in accordance with the Declaration of Helsinki and Guidelines for Good Clinical Practice. All patients gave written informed consent. A general graphical representation of the Materials and methods section is shown in Supplementary Fig. 1.

## Results

During the course of the LyMa-101 study, 86 patients were enrolled and 67 patients had MRD assessment at the end of induction, corresponding to the primary endpoint, quantified by quantitative PCR. Baseline FDG-PET/CT data were available for 59 patients of them. These patients did not statistically differ than the rest of LyMa-101 population in terms of baseline characteristics, follow-up and outcome (Table 1). In our cohort, 11 patients (19%) were MRD-positive and 48 (81%) reached MRD negativity by the end of induction, before ASCT.

**Table 1:** Demographical and bio-clinical characteristics.

Characteristics	Study population (n = 59)	LyMa-101 population (n = 86)
Median age (years)	54.9 (32–65)	58 (51–62)
Sex		
Male	41 (69%)	63 (73%)
Female	18 (31%)	23 (27%)
Performance status (ECOG)		
0	39 (66%)	55 (64%)
1	19 (32%)	27 (31%)
2	1 (2%)	4 (5%)
MIPI score		
Low risk	34 (57%)	47 (55%)
Intermediate risk	15 (25%)	24 (28%)
High risk	10 (17%)	14 (16%)
Ki-67*		
> 30%	17 (29%)	29 (34%)
Blastoid variant	11 (19%)	15 (17%)
Ann Arbor stage		
II	0	2 (2%)
III	3 (5%)	6 (7%)
IV	56 (95%)	78 (91%)

**Figure 1:**
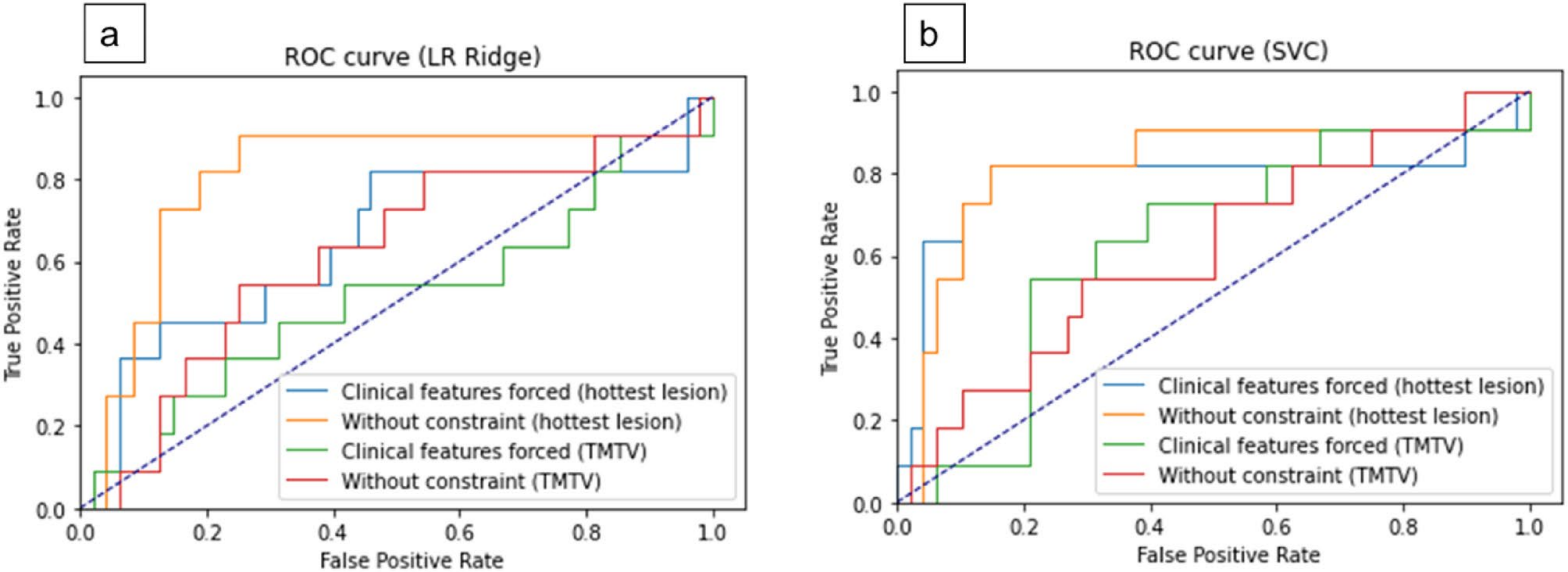
ROC curves for LR Ridge (**a**) and for SVC (**b**) according to the disease area considered for RF computation (hottest lesion or TMTV).

**Figure 2:**
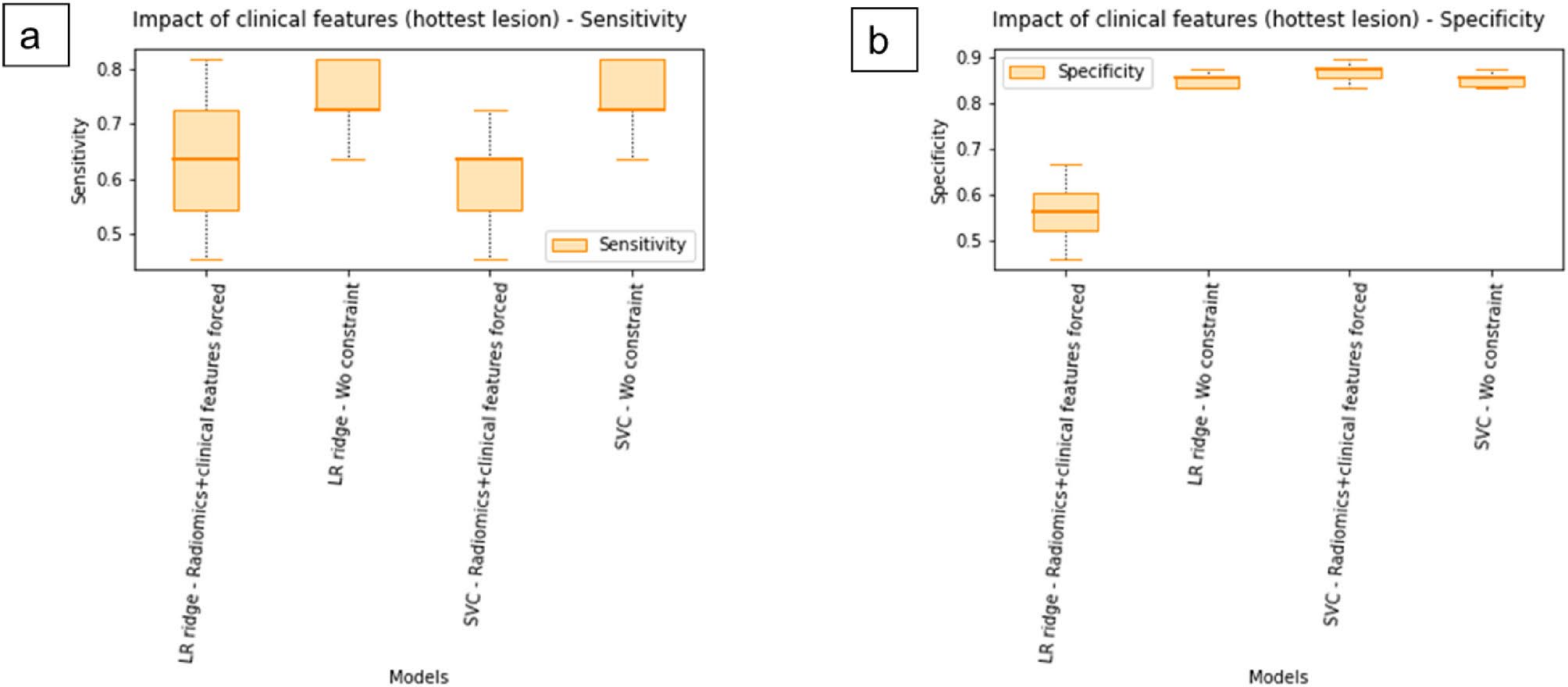
Impact of the use of clinical features on sensitivity (a) and specificity (b) for LR Ridge and SVC (disease area: hottest lesion).

LR Ridge with or without constrained clinical and biological data showed AUCs of up to 0.63 and 0.82 for negative vs positive MRD in the lesion with the highest uptake and 0.53 and 0.64 in the TMTV (Fig. 1a). SVC with or without constrained clinical and biological data showed AUCs of up to 0.73 and 0.80 for negative vs positive MRD in the lesion with the highest uptake and 0.51 and 0.62 in the TMTV (Fig. 1b). Furthermore, the constrained use of clinical and biological features showed a clear loss in sensitivity and specificity for the prediction of MRD status before ASCT, regardless of the machine learning model (Fig. 2). Of note, no conventional PET parameters remained among the ten most important PET-derived features selected after MRMR.

## Discussion

Despite the limited sample size, a number of relevant conclusions can be drawn from our findings. First, this study reinforced the previously made hypothesis that the prognosis of the disease in MCL patients is linked to the most aggressive contingent^11^, within the lesion with the highest uptake, the performance of the models based on the TMTV appearing inferior. Despite a prognostic value described in other non-Hodgkin lymphomas^12^ and in one MCL study^13^, FDG-PET/CT calculation of total tumor volume load does not seem to be of interest in this subtype of lymphoma. This might be explained by the frequent splenic involvement in MCL, sometimes massive, and which is not systematically associated with a poorer prognosis^10^ but responsible of important metabolic volumes and thus large inter-individual variability. Second, as already demonstrated^5,6,14^, these data plead for the importance of RF in MCL patients. In our work, whether the models were operator-supervised with imposed clinical features commonly known for their high prognostic value, or whether the models were free to determine all features based on their importance, all selected features were RF. They outperformed classical FDG-PET/CT features and were of higher importance than all clinical and biological features. These results are slightly different from the data published by Mayerhoefer et al. in which the combination of ECOG performance status, WBC, LDH level and Ki-67 index to the radiomic signature significantly increased 2-years progression free survival (PFS) prediction^6^. In their study, RFs were extracted from the TMTV which explains probably the discrepancies. It should be noted, however, that this study considered PFS as an endpoint, whereas we assessed the impact of these features on MRD, an indirect surrogate of survival.

Another particularity of our work regarding the literature is the use of bone marrow MRD rather than PFS or overall survival as our clinical endpoint, in contrast to many lymphoma studies that focused on prognostic features. This approach was selected in the LyMa-101 study because MCL is a relatively rare subtype of lymphoma, and thus more limited sample size and shorter follow-up times can be employed to achieve a suitable number of events. Several phase 2 or 3 prospective trials have reported the correlation between MRD and PFS supporting its use before ASCT for treatment management of MCL patients as a surrogate marker^2,3^ for long-term outcome. Moreover, MRD residual disease negativity in bone marrow is more difficult to achieve than in peripheral blood, allowing for a rigorous evaluation of treatment efficacy.

The main limitation of the current work is obviously the lack of an external test set to confirm the reported results and minimize the risk of overfitting. Given the relatively rare disease incidence, an LOOCV was used to account for the small dataset of the Lyma-101 study. The intuition reported in the current work needs to be confirmed by an external dataset using the same therapy strategy. Finally, the small number of patients per center involved in this multicentric study prevented the use of harmonisation techniques such as ComBat-like approaches^15^. Therefore, it was not considered in this study.

In conclusion, our results demonstrated the predictive value of initial FDG-PET/CT on bone marrow MRD after induction in the Lyma-101 study and the importance of FDG-PET/CT RF extracted from the lesion with the highest uptake compared to clinical and laboratory parameters. Although these metrics are not routinely calculated, these data again show the value of FDG-PET imaging in MCL and the likely need to integrate radiomics into a personalized multi-technology treatment-risk profiling composite score. Further prospective investigations are warranted to confirm these observations.

### Supplementary Information

Supplementary Figure 1.

## Data Availability

The datasets generated during and/or analysed during the current study are available from the corresponding author on reasonable request.
