# Supplementary material for: Performance of baseline FDG-PET/CT radiomics for prediction of bone marrow minimal residual disease status in the LyMa-101 trial
Source: Sci Rep. 2023 Oct 24;13:18177. doi: 10.1038/s41598-023-45215-y (PMC10598231; doi:10.1038/s41598-023-45215-y)
Supplement: Supplementary file 1 — Supplementary Figure 1. [file 41598_2023_45215_MOESM1_ESM.docx]

**Supplementary Data**


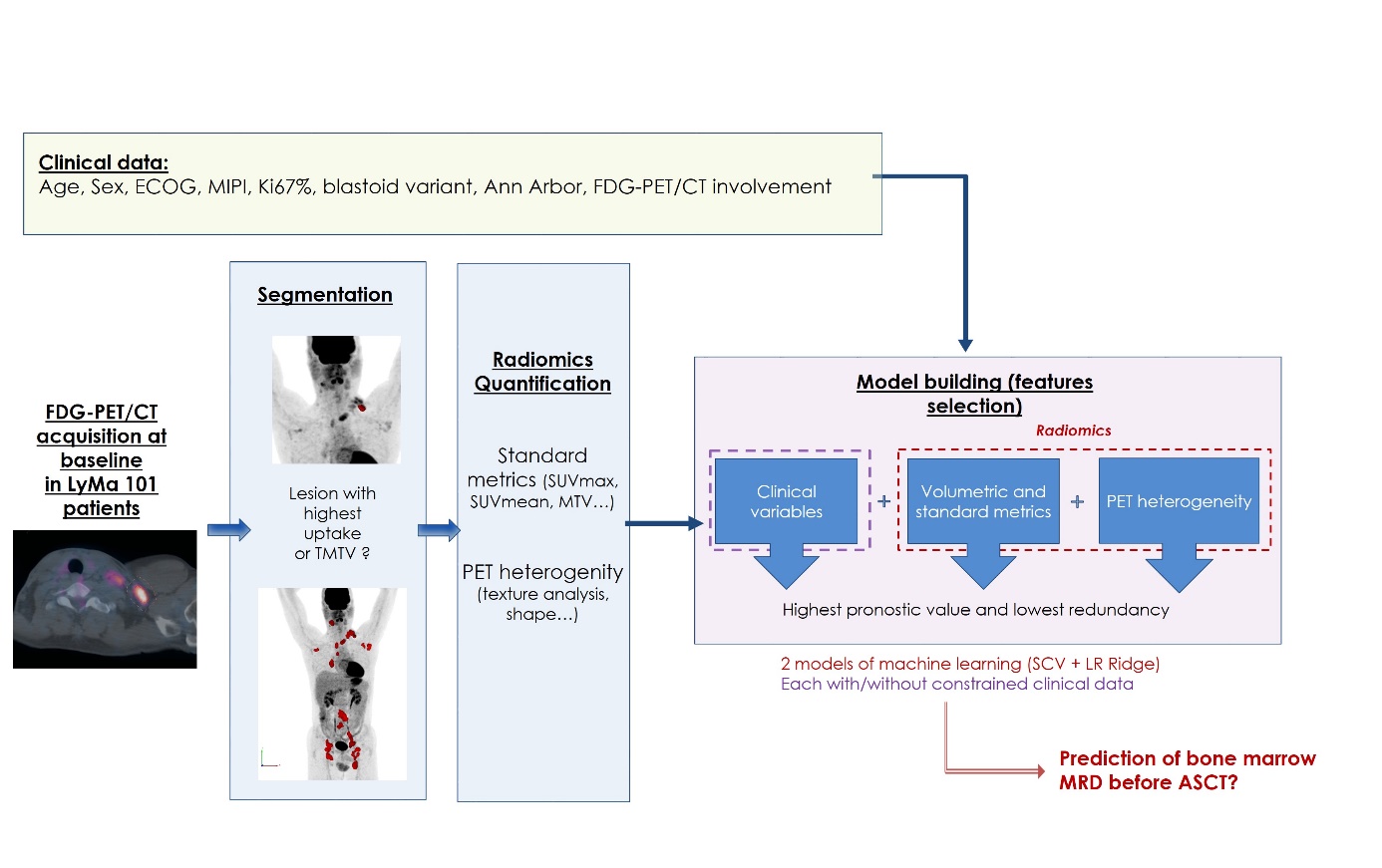


Supplementary Figure 1 : General graphical representation of the Materials and methods section
